# Supplementary material for: Splice-Junction-Based Mapping of Alternative Isoforms in the Human Proteome
Source: Cell Rep. Author manuscript; Available in PMC 2020 Jan 15. (PMC6961840; doi:10.1016/j.celrep.2019.11.026)
Supplement: 2 [file NIHMS1546469-supplement-2.zip › DF1/PXD006675/RightAtrium/RightAtrium_29_SRRM1_KVELSESEDK.pdf]

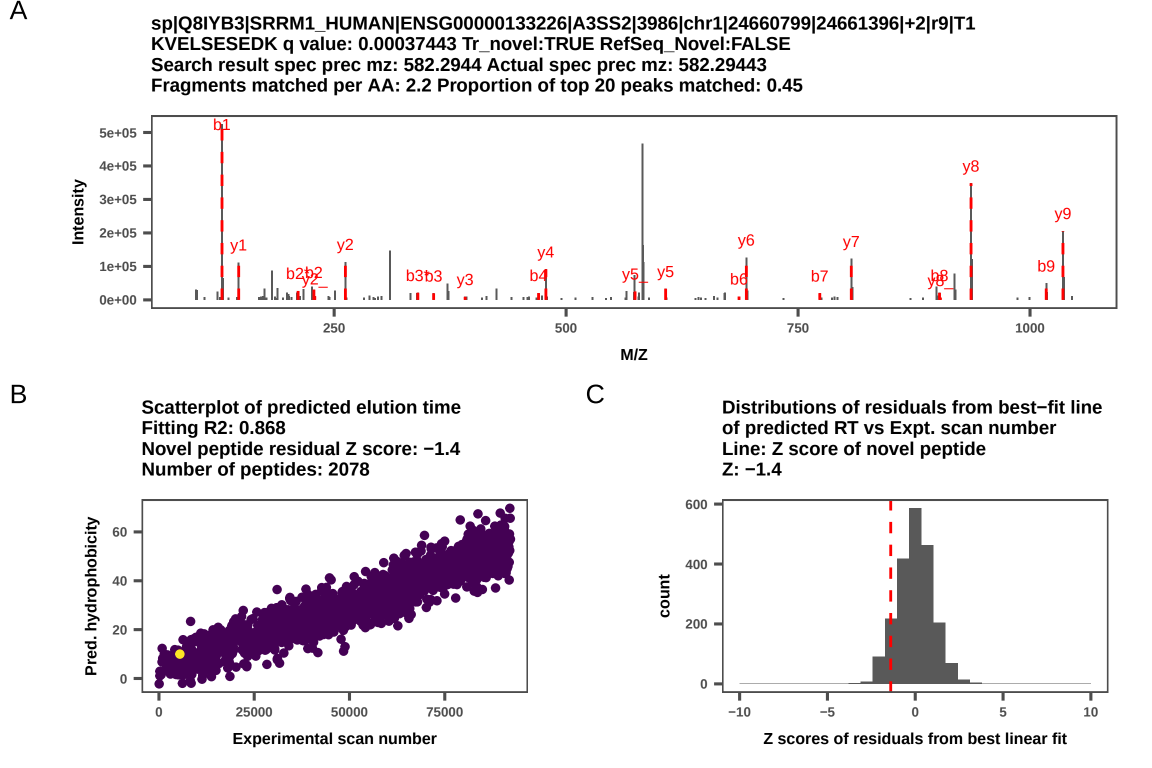
A
sp|Q8IYB3|SRRM1_HUMAN|ENSG00000133226|A3SS2|3986|chr1|24660799|24661396|+2|r9|T1
KVELSESEDK q value: 0.00037443 Tr_novel:TRUE RefSeq_Novel:FALSE
Search result spec prec mz: 582.2944 Actual spec prec mz: 582.29443
Fragments matched per AA: 2.2 Proportion of top 20 peaks matched: 0.45
Intensity
5e+05
4e+05
3e+05
2e+05
1e+05
0e+00
b1
y1
b2
b2
y2_
y2
b3
b3
y3
b4
y4
y5_
y5
b6
y6
b7
y7
b8
y8_
y8
b9
y9
250
500
750
1000
M/Z
B
Scatterplot of predicted elution time
Fitting R2: 0.868
Novel peptide residual Z score: −1.4
Number of peptides: 2078
Pred. hydrophobicity
0
20
40
60
0
25000
50000
75000
Experimental scan number
C
Distributions of residuals from best−fit line
of predicted RT vs Expt. scan number
Line: Z score of novel peptide
Z: −1.4
count
0
200
400
600
−10
−5
0
5
10
Z scores of residuals from best linear fit
